# Quantifying biomolecular hydrophobicity: Single molecule force spectroscopy of class II hydrophobins

**DOI:** 10.1016/j.jbc.2021.100728

**Published:** 2021-04-30

**Authors:** Arja Paananen, Sabine Weich, Géza R. Szilvay, Michael Leitner, Kirsi Tappura, Andreas Ebner

**Affiliations:** 1 Industrial Biotechnology and Food, VTT Technical Research Centre of Finland Ltd, Espoo, Finland; 2 Department of Applied Experimental Biophysics, Institute of Biophysics, Johannes Kepler University Linz, Linz, Austria

**Keywords:** single molecule force spectroscopy, SMFS, hydrophobic interaction, hydrophobin, HFBI, *Trichoderma reesei*, class II hydrophobin, AFM, atomic force microscopy, APTES, aminopropyltriethoxysilane, FDC, force distance cycle, NCys-HFBI, a genetically engineered HFBI variant containing cysteine residue in the linker attached to the N terminus, NHS-PEG-Mal, O-[N-(3-maleimidopropionyl)aminoethyl]-O′-[3-(N-succinimidyloxy)-3-oxopropyl]heptacosaethylene glycol, PDF, probability density function, SMFS, single molecule force spectroscopy, TEA, triethylamine

## Abstract

Hydrophobins are surface-active proteins produced by filamentous fungi. The amphiphilic structure of hydrophobins is very compact, containing a distinct hydrophobic patch on one side of the molecule, locked by four intramolecular disulfide bridges. Hydrophobins form dimers and multimers in solution to shield these hydrophobic patches from water exposure. Multimer formation in solution is dynamic, and hydrophobin monomers can be exchanged between multimers. Unlike class I hydrophobins, class II hydrophobins assemble into highly ordered films at the air–water interface. In order to increase our understanding of the strength and nature of the interaction between hydrophobins, we used atomic force microscopy for single molecule force spectroscopy to explore the molecular interaction forces between class II hydrophobins from *Trichoderma reesei* under different environmental conditions. A genetically engineered hydrophobin variant, NCys-HFBI, enabled covalent attachment of proteins to the apex of the atomic force microscopy cantilever tip and sample surfaces in controlled orientation with sufficient freedom of movement to measure molecular forces between hydrophobic patches. The measured rupture force between two assembled hydrophobins was ∼31 pN, at a loading rate of 500 pN/s. The results indicated stronger interaction between hydrophobins and hydrophobic surfaces than between two assembling hydrophobin molecules. Furthermore, this interaction was stable under different environmental conditions, which demonstrates the dominance of hydrophobicity in hydrophobin–hydrophobin interactions. This is the first time that interaction forces between hydrophobin molecules, and also between naturally occurring hydrophobic surfaces, have been measured directly at a single-molecule level.

Hydrophobins are fungal proteins possessing extraordinary surface activity and properties that have fascinated scientists for decades (1, 2, 3). The application potential of hydrophobins, for example, as foam and emulsion stabilizers, dispersants of solids, and in immobilization of bioactivity on surfaces, is widely acknowledged (4) and makes them very interesting molecules to study. Hydrophobins are relatively small (7–15 kDa) surface active proteins produced by filamentous fungi. Their biological roles involve surface adhesion and as coating and protective agents. They are very efficient in lowering the surface tension of water, which is vital for the hyphal growth through the air–water interface (4). Hydrophobins are divided into two classes, class I and class II based on their hydropathy plots and how they assemble at the air–water interface. Class I hydrophobins form amyloid-like fibrils with strong intermolecular interactions, whereas class II hydrophobins assemble into highly ordered films. Hydrophobins are especially known for forming strong interfacial films (1, 2). The superior surface elasticity of hydrophobin films (5, 6) originates from the ordered self-assembled structure at fluid interfaces (7, 8, 9, 10, 11). This property has recently been utilized in bilayer studies of hydrophobin films by microfluidic approach (12), where bilayers’ adhesion energy was determined. Also, the pH of the surroundings plays a role; it has been shown that changes in pH alter the structure and elasticity of the hydrophobin film by reorienting hydrophobin molecules and subsequently affecting the interaction between the hydrophobins at the air–water interface (13, 14). The amphiphilicity of hydrophobins originates from their unique and very rigid structure, which is fixed by four intramolecular disulfide bonds (15). Instead of having hydrophobic residues buried inside the protein structure, hydrophobins contain a distinct hydrophobic patch on one side of the molecule (Fig. 1). This hydrophobic patch seems to dominate the behavior and function of hydrophobins.

**Figure 1:**
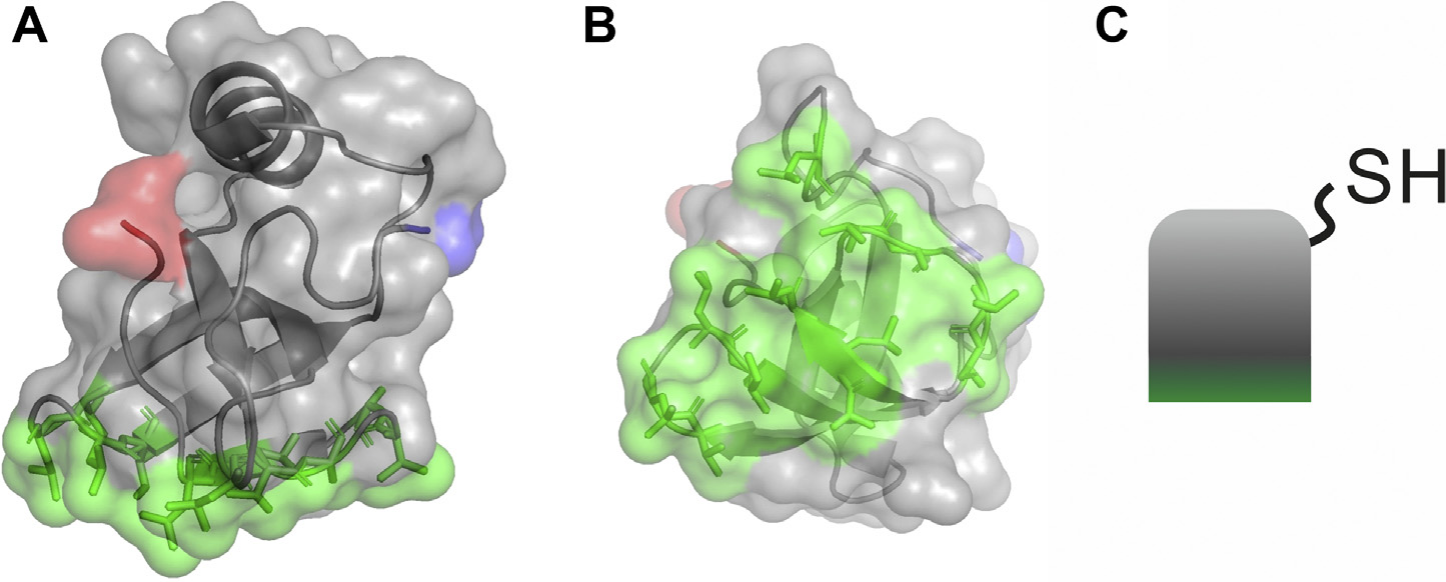
**X-ray crystal structure of wildtype HFBI (Protein Data Bank ID****2FZ6****)** (15)**.** In a side view (*A*) and a bottom view (*B*) of HFBI the N terminus is shown in *blue*, C terminus in *red*, and the hydrophobic patch in *green*. The protein structures were produced with PyMol (53). A schematic picture of the NCys-HFBI molecule is shown in (*C*).

Hydrophobins are very soluble in water, and it has been shown that they form multimers to minimize the area of the hydrophobic patch exposed to water (16). This tendency of hydrophobic units to associate in aqueous solution is natural behavior and originates from van der Waals forces (17, 18). Hydrophobin multimers in solution are dynamic, and they are able to disassemble and reassemble continuously (19, 20). Hydrophobic interaction is clearly involved in the interaction between hydrophobins, but the strength of this interaction is not known.

The atomic force microscopy (AFM)-based single molecule force spectroscopy (SMFS) (21, 22) has been developed to reveal the energy landscape underlying a biological interaction (23). This is realized by single molecule pulling experiments where a force is applied on a biomolecular complex resulting in lowering the energy barrier. Measurements at different pulling velocities onto the same system yield the dependence of the complex rupture force on the rate of the applied force load. This allows, according to the Bell Evans model (24), calculation of kinetic and structural parameters, namely, the kinetic dissociation rate k_off_ as well as the width of the energy barrier x_β_. SMFS allows monitoring these structural and kinetic parameters of interactions on the molecular level. Nevertheless, classical biomolecular complexes do not rely on hydrophobicity alone. Other noncovalent weak interactions ranging from hydrogen bonding to electrostatic also play a central role.

In this work influence of the environment, especially the nature of the solvent on the homophilic hydrophobin interaction was investigated in detail by measuring the interaction force between hydrophobins at different conditions using SMFS. We used NCys-HFBI molecules, which are engineered HFBI (class II hydrophobin from *Trichoderma reesei*) variants containing a cysteine residue at the end of a 11-amino-acid-long linker at the N terminus (19). Hence, the variant molecules can be covalently attached to surfaces from the hydrophilic side of the molecule leaving the hydrophobic side free to interact and with some flexibility *via* the built-in linker. For tethering hydrophobins to the AFM tip, additionally a PEG linkage was used to ensure sufficient motional freedom overcoming steric hindrance in complex formation (25). This was the very first time that interaction forces between naturally occurring hydrophobic surfaces have been measured directly at a single molecule level.

## Results

### Functionalized surfaces

**Figure 2:**
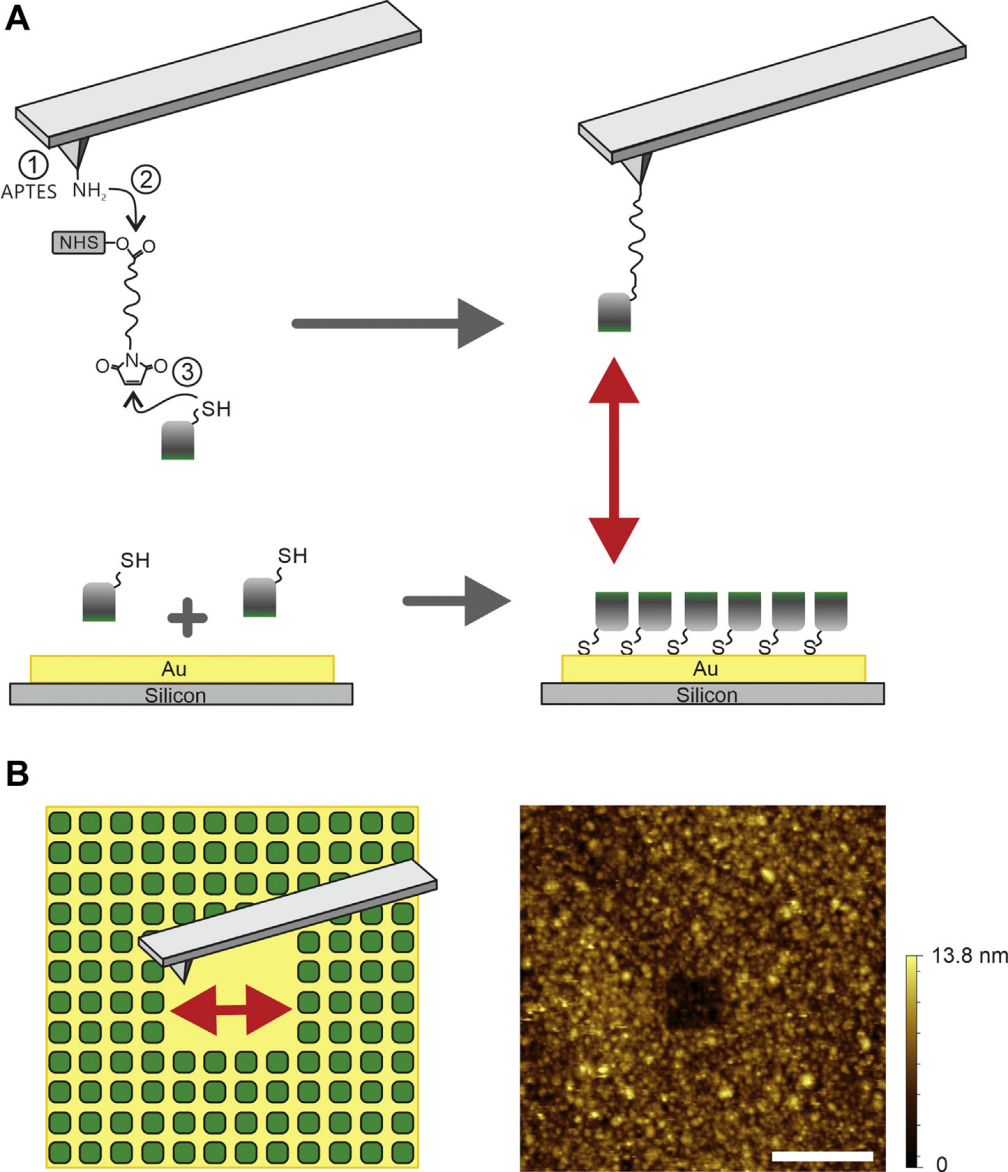
**Tip and surface functionalization.***A*, coupling scheme of tip functionalization with NCys-HFBI. (1) In a first step reactive amino residues on the AFM tip are introduced by amino functionalization. (2) The hetero-bifunctional PEG cross-linker NHS-PEG-Mal (54) is coupled. (3) NCys-HFBI, an engineered variant of HFBI containing a 11-amino-acid-long linker with a Cys residue in the N terminus (19), is attached to the maleimide end of the linker *via* its cysteine residue. The cysteine residue is also used for attaching the hydrophobins to a gold surface. *B*, to proof the NCys-HFBI immobilization on gold a scratching experiment was performed after actual force measurements and blocking experiments. For this, NCys-HFBI was incubated on a cleaned gold substrate, and after this, a bilayer was formed by dimerization of wildtype HFBI *via* their hydrophobic parts. A small area was scratched by a sharp AFM tip with high indentation force and imaged afterward with lowered force (*B*, *left*). The AFM image (*B*, *right*) shows a height difference of ∼6 nm corresponding to the height of two hydrophobin molecules. Scale bar, 1 μm. AFM, atomic force microscopy.

AFM-based biological imaging is an excellent tool to investigate structures and morphological details down to the nanometer scale. Furthermore, the AFM-based technique SMFS allows one to investigate weak (noncovalent) interactions of single individual biomolecules. To explore hydrophobic interactions, especially those of hydrophobins (Fig. 1) at molecular level, tight and well-defined tethering of the two binding partners both on the solid support and on the sensor is required. More precisely, one hydrophobin molecule has to be immobilized on the outer tip apex of the sensing AFM tip, whereas the other hydrophobin has to be tethered to the solid support. For sensor tip functionalization we used the well-established aminopropyltriethoxysilane (APTES) amino-silanization protocol (Fig. 2*A* ①), followed by covalent coupling of the thiol-reactive heterobifunctional PEG cross linker O-[N-(3-maleimidopropionyl)aminoethyl]-O′-[3-(N-succinimidyloxy)-3-oxopropyl]heptacosaethylene glycol (NHS-PEG-Mal) (Fig. 2*A* ②). Finally, hydrophobins exhibiting a sulfhydryl group (NCys-HFBI) were covalently coupled to the maleimide residue of the PEG linker (Fig. 2*A* ③). The N terminus is located in the hydrophilic side of the molecule (Fig. 1*C*), and hence, the hydrophobic side is left to interact freely with other molecules. The reactive thiol group of the cysteine mutant furthermore allowed a straightforward coupling of the proteins to a solid gold support (*i.e.*, gold-coated quartz chip of quartz crystal microbalance). The reaction between the free thiol group in NCys-HFBI and gold surface ensured a firm attachment of a protein monolayer on the sample support.

AFM imaging on the hydrophobin-coated gold was performed to ensure the formation of a dense protein layer on the substrate. This was studied after the actual force spectroscopy measurements and specificity proof experiments. A full coverage of the gold surface by proteins was observed. Scratching experiments using a high indentation force at a smaller scan area (as drawn in Fig. 2*B*, left), followed by topographical imaging of the area after performing the scratching protocol, clearly proved the formation of a bilayer of hydrophobin proteins (Fig. 2*B*, right). The height difference between the upper level of the outer hydrophobin coated area and the substrate was 5.8 ± 1.3 nm, which is in accordance with the expected height of a hydrophobin bilayer (8, 26).

### Interaction forces between single hydrophobin molecules

**Figure 3:**
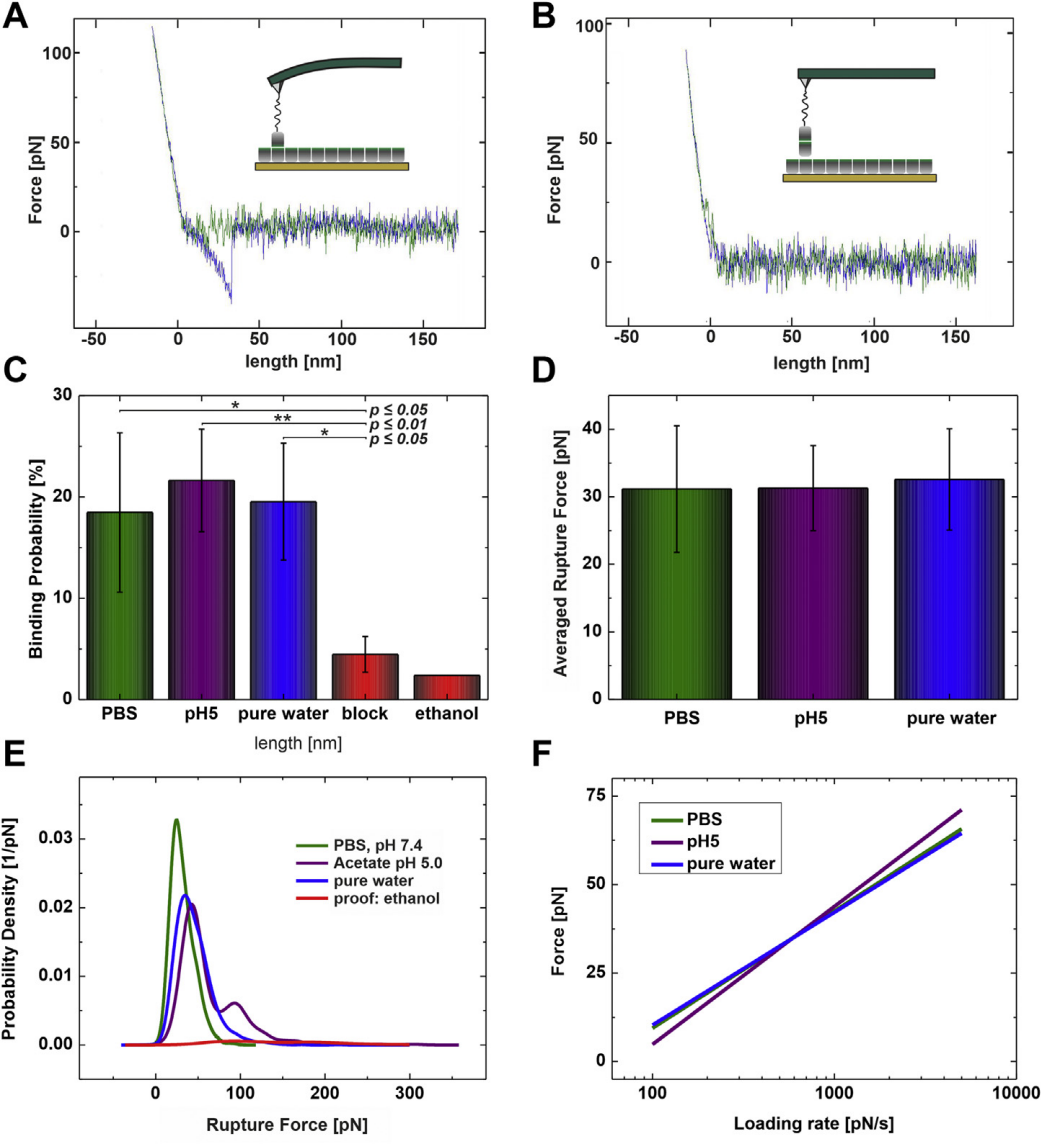
**Force measurements and calculated results**. *A*, a typical force distance cycle (FDC) showing molecular hydrophobin–hydrophobin interaction. The HFBI-functionalized tip is approached (*green line*) to the surface functionalized with a monolayer of NCys-HFBI. After reaching the surface, further approach results in an upward bending of the cantilever. In the retraction part (*blue line*) the cantilever reaches its resting position at the point of contact. In case of formation of a hydrophobin dimer further retraction results in an additional downward bending until the pulling force is stronger than the homophilic interaction causing a sudden rupture. *B*, FDC of the same arrangement but with a blocked tip. *C*, dependence of the binding probability on environmental conditions. In pH 7.4 and pH 5.0 buffers as well as in pure water the mean binding probability is ∼20%. In contrast, both the addition of 70% ethanol and the blocking of the binding site by addition of free hydrophobins significantly reduce the binding probability to 2.5% to 5%. *D*, averaged rupture forces at the same loading rates for different environmental conditions. *E*, probability density function of the homophilic hydrophobin interaction at different conditions (*green line*, PBS; *purple line*, acetate buffer adjusted to pH 5; *blue line*, ultrapure water; *red line*, specificity proof in 70% ethanol). *F*, loading rate dependence of the unbinding force of the homophilic hydrophobin interaction at different environmental conditions fitted with the maximum likelihood approach.

We used SMFS to investigate the interaction forces between single homophilic hydrophobin–hydrophobin interactions. For this, a monomolecular hydrophobin AFM sensing tip was generated as described in Materials and Methods. As the corresponding binding partner, *i.e.*, another hydrophobin molecule, we used a dense hydrophobin layer that was immobilized on the gold substrate. In so-called force distance cycles (FDCs) the interaction forces were monitored; a typical FDC exhibiting a molecular hydrophobin–hydrophobin interaction is shown in Figure 3*A*. As a result, from FDCs, the deflection of the cantilever is shown as a function of distance between the tip and the sample surface. The hydrophobin tethered tip was approached to the hydrophobin functionalized surface, whereas both tip-surface distance as well as the cantilever bending force was monitored (Fig. 3*A*, green curve). At the point of contact, further approaching results in a linear upward bending of the cantilever. The approaching period stops automatically if a previously set indentation force limit is reached. To avoid any degenerative or denaturing effects on the hydrophobins we set this limit to a very low value, ∼100 pN. In the retraction period, shown as the blue curve in Figure 3*A*, the bending decreases by removal of the tip from the sample surface. Loosing contact results in reaching the resting position of the cantilever since no force is acting. Nevertheless, the contact time between the sample surface and the tip allowed homophilic hydrophobin–hydrophobin complex formation. In this case, further retraction of the tip yields an additional but downward bending of the cantilever. The deflection of the cantilever in the moment of rupture gives, according to Hooks law, the rupture (or unbinding) force F(u) of a single hydrophobin–hydrophobin interaction at the applied conditions. The distance between the point of contact and the rupture event corresponds to the size of the interacting molecules and stretching of the PEG linker. In contrast, in the specificity proof experiment with a blocked tip, as schematically drawn as inset in Figure 3*B*, the approach (green) and withdrawing (blue) curves were identical and the downward bending disappeared (Fig. 3*B*). The interaction forces of the binding events were analyzed, and the probability density functions (PDFs) (27) were generated (Fig. 3*E*). The maximum value in PDF corresponds to the most probable unbinding force of the hydrophobin–hydrophobin interaction.

By performing SMFS with the single class II hydrophobins tethered on the tip as well as the covalently immobilized ones on the surface, we could show a rupture force of ∼31 pN for the homophilic hydrophobin interaction at a loading rate of 500 pN/s. We investigated this hydrophobin–hydrophobin interaction at different conditions, namely, in pure water, PBS buffer (pH 7.4), and Na-acetate buffer pH 5. Under all the three conditions, both complex formation and rupture behavior showed similar results. In detail: the binding probability (*i.e.*, the number of formed complexes divided by the total number of performed FDCs at the same environmental and physical conditions) was 18.47 ± 7.85% in PBS pH 7.4, 21.63 ± 5.07% at pH 5 in acetic acid buffer, and 19.53 ± 5.77% in pure water, thus showing no significant difference (Fig. 3*C*). In total, 14,106 FDCs within eight datasets have been recorded in PBS, 14,967 FDCs in acetate buffer at pH 5 within ten datasets, and 23,096 FDCs within ten datasets in pure water. Furthermore, the most probable rupture forces, derived from about 600 specific unbinding events each and averaged over the same loading rates, were very similar for all the three environmental conditions (Fig. 3*D*): 31.14 ± 9.35 pN in PBS, 31.29 ± 6.29 pN in acetic acid buffer, and 32.57 ± 7.50 pN in pure water.

The specificity of these interactions was tested by two independent measurements. It is known (19) that a solution of ethanol:water in a ratio of 7:3 prevents the dimerization of hydrophobins. Thus, we performed the experiments at the same conditions recording about 2000 FDCs in a 7:3 mixture of ethanol and water resulting in a significant decrease of the probability of complex formation down to 2.4% (Fig. 3*C*). In the second specificity proof experiment performed in PBS, we added free hydrophobin molecules to the measurement configuration to allow complex formation both on the tip and on the surface to block the hydrophobic patches (see also Fig. 3*B*). As a result of recording and analyzing about 4000 FDCs within four datasets, again, a clear drop of the probability of complex formation down to 4.47 ± 1.76% was observed (Fig. 3*C*). A student *t* test proved the significance of the differences before and after the blocking for all three liquids, as shown in Figure 3*C*. It should be mentioned that binding probabilities of 5% or lower are common for any receptor ligand system and have their origin most probably in nonspecific adhesion of the AFM tip on the sample surface. Summing up, we could clearly demonstrate that the majority of observed rupture events are caused by the homophilic interaction of two hydrophobin molecules. Figure 3*E* shows the probability density function of the very same ligand receptor pair at comparable loading rates in PBS (green line), in acetate buffer pH 5 (purple line), and in pure water (blue line) resulting in the binding probabilities of 17.4%, 20.9%, and 19.6%, respectively, with a pronounced maximum in the unbinding forces as described previously. In contrast, the same measurement performed in 70% ethanol (Fig. 3*E*, red line) resulted in a 10-fold lower binding probability (2.4%), whereas no clear maximum in the unbinding force was observable.

### Energy landscape of the hydrophobin–hydrophobin interaction

Although this forced dissociation is of high interest *per se*, since it allows quantifying molecular forces required to rupture a pair of hydrophobins under a given force load, further information on the molecular scale can be gained by applying SMFS to the system. Thus, we performed the same experiment at different loading rates by varying the pulling velocity from 50 to 2000 nm/s resulting in a loading rate range over two orders of magnitude (Fig. 3*F*). Fitting the data according to Bell Evans model (24, 28) using a maximum likelihood approach (as described previously) (29) yielded kinetic and structural data of the interaction. As a result, the homophilic hydrophobin interaction showed in all the cases just one single energy barrier with a complex dissociation constant k_off_ of 3.61 ± 0.015 s^−1^ and a width of the energy barrier x_β_ of 2.85 ± 0.043 Å for PBS buffer, 4.44 ± 0.020 s^−1^ and 2.42 ± 0.043 Å for the interaction at pH 5 in acetic acid buffer, and 3.43 ± 0.014 s^−1^ and 2.96 ± 0.039 Å for ultrapure water. These slight differences are also visible in the loading rate plot as shown in Figure 3*F*. Of interest, in all the different environments the homophilic binding behavior appears very similar, which may be attributed to the stable structure of hydrophobins and/or the lack of electrostatic participation within the dimerization process.

## Discussion

Hydrophobins are fascinating proteins owing to their unique structure: instead of burying hydrophobic residues inside the protein structure, these proteins contain four disulfide bridges that stabilize a nanometer-sized hydrophobic patch on one side of the molecule. In this work, we wanted to gain an insight about the hydrophobicity and strength between two natural biomolecular hydrophobic surfaces. For this we used an SMFS setup to quantitate intermolecular forces. We rationalized that such measurements could be feasible with site-specific tethering of hydrophobins onto surfaces. A hydrophobin variant NCys-HFBI, which contains an additional free cysteine residue on the hydrophilic side of the molecule, would enable the attachment of the protein in a controlled orientation on an AFM probe and on a surface and, consequently, a direct SMFS measurement between hydrophobic patches.

Understanding of hydrophobicity has been a well-studied topic for a long time. The early studies by Kauzmann (30) and Tanford (31, 32) initiated active exploration of hydrophobicity in order to understand association, adsorption, and self-assembly processes at molecular length scale. The first pioneering measurements by Israelachvili *et al.* (33, 34) based on the surface force apparatus (35) showed that hydrophobic interaction has similar range, but is an order of magnitude stronger than van der Waals interaction, and the interaction decays exponentially in the range of 0 to 10 nm. The later studies showed that observed long-range interactions seemed to depend greatly on the force-measuring techniques and methods for surface hydrophobization, and that the true hydrophobic interaction is short-ranged (<10 nm) (36, 37). More recently Ducker and Mastropietro (38) have examined closely the published interaction studies related to hydrophobic interaction and reported that the decay length of a hydrophobic force is in the range of 0.3 to 1.0 nm. In addition, Stock *et al.* (39) have reported based on their molecular scale force spectroscopy experiments and molecular dynamics simulations that hydrophobic amino acids in a peptide with increasing amount of hydrophobic amino acids interact independently and additively with a hydrophobic surface.

To explore the interaction between hydrophobin molecules the solution behavior of HFBI and HFBII hydrophobins has been investigated. The Förster resonance energy transfer experiments reported by Szilvay *et al.* (19) showed that class II hydrophobins HFBI and HFBII form dimers and multimers by hydrophobic interaction and that already 25% ethanol reduced the hydrophobin–hydrophobin association, but higher salt concentration enhanced the interaction. Similar conclusions were reported also from small- and wide-angle X-ray scattering experiments for HFBI and HFBII (7, 16, 40, 41), where in addition, early solution structures for hydrophobin assemblies were reported. These findings demonstrate the importance of hydrophobic interactions between hydrophobins. Later, stopped-flow fluorescence measurements (20) of HFBII and HFBI indicated that multimer formation of hydrophobins is a relatively slow process, but not as slow process as to be a rate-limiting step for interfacial self-assembly, and that multimer formation is reversible, there is continuous exchange of molecules between multimers. Recently, interaction forces between HFBI hydrophobin layers have been investigated to get more insight to the hydrophobicity. Goldian *et al.* (42) coated mica surfaces with HFBI and measured interaction forces with surface force balance in aqueous environment. The results showed the hydrophobic nature of the interaction between hydrophobin layers for the relatively large contact area containing many individual hydrophobin molecules.

The strength of interaction between hydrophobin molecules in multimers has remained elusive, though. Recently, interactions between single hydrophobin molecules and hydrophobic surfaces have been investigated. Griffo *et al.* (43) reported on force spectroscopy studies of modular resilin protein, where one end of the fusion protein comprised HFBI. They obtained values for rupture forces between the hydrophobic parts of the molecule, which can be the hydrophobic patch of HFBI, and the hydrophobic tip surface in the range from 50 to 200 pN at pH 5 and concluded the force to be similar to previous force studies between hydrophobic surfaces and proteins. Li *et al.* (44) have reported interaction forces between graphite (highly oriented pyrolytic graphite) surfaces and hydrophobin (HFBI) molecules attached to the AFM tip *via* a flexible linker in PBS (pH 7.4). The most probable rupture force in this more straightforward setup was approximately 70 pN at a loading rate of 500 pN/s (44). They reported also that detachment of a single hydrophobin molecule from a hydrophobin layer requires even more force, which indicates that also the interactions between the hydrophilic sides of hydrophobins are important for the functionality of hydrophobins in films and coatings.

In this work, a unique SMFS configuration allowed us to measure the most probable rupture forces for unbinding hydrophobin dimers. The short linker anchoring hydrophobin molecules to the gold surface allowed some freedom for hydrophobins to interact optimally with the tip-bound hydrophobin molecule. Hence, we expect that the strongest interactions observed in the experiments originated from a single gold surface–bound hydrophobin interacting with the tip-bound hydrophobin resembling a dimer interaction in solution, where the hydrophobic patches are best shielded from the surroundings. The determined rupture force values averaged over the same loading rate range were very similar for all the different environmental conditions (Fig. 3, *D* and *F*). Furthermore, when comparing the binding probabilities and most probable rupture forces, both the complex formation and rupture behavior showed similar results in all the liquids. In addition, in all the cases, the hydrophobin–hydrophobin interaction could be eliminated by adding free soluble hydrophobins, or in water also by adding ethanol, proving the specificity of the interaction and hydrophobic nature.

The obtained interaction forces between single hydrophobin molecules are in the comparable range but smaller than the corresponding values between single hydrophobins and the hydrophobic surfaces described above (Griffo *et al.* (43) pH 5, Li *et al.* (44) pH 7.4). The differences can be explained by chemical and mechanical differences between hydrophobic surfaces and the hydrophobic patch of hydrophobin molecules. According to the theoretical work conducted by Acharya *et al.* (45) and Ma *et al.* (46), protein surfaces possess heterogeneity in hydrophobicity and surface roughness, *i.e.*, the hydrophilic amino acids in the vicinity of hydrophobic residues may disturb the hydrophobic interaction. Also, the conformation of the protein surface adapts better to the surroundings during the interaction in solution than on a solid surface. In the HFBI X-ray crystal structure (15) two monomers are associated together through the hydrophobic patch in an overlapping fashion so that the hydrophobic patches are only partially covered. The structure indicates that this might be a preferred assembly configuration for association.

In this work, we have quantified the interaction forces between HFBI molecules and compared the results with the force values between HFBI and hydrophobic surfaces. The results show that regardless of changes in pH or in ionic strength the interaction is weaker between HFBI molecules than between HFBI and a hydrophobic surface. This finding has an important biological meaning: in solution the protein prefers to be associated, but in the vicinity of an interface the protein prefers to attach to the interface. This means, similar to detergent micelles, a weaker interaction between hydrophobin monomers enables surface activity while allowing for solubility. As the difference in interaction forces serves an important functional role, the interprotein association is likely determined by specific interactions.

Filamentous fungi are known to grow on organic substrates where they degrade the material, such as wood, for nutrients. Water is strongly involved in fungal growth, though. It has been speculated that dimerization and multimerization of hydrophobins in solution are intermediates for hydrophobins to protect their hydrophobic patches from water before attaching to surfaces or interfaces (40). The studied class II hydrophobin molecules are relatively rigid and robust proteins, as their compact structure interlocked by four disulfide bridges does not unfold very easily (15). The interacting hydrophobic patch located on one side of the hydrophobin molecule forms a relatively flat surface, and there is no involvement of great conformational changes upon association. The strength of the interaction between ligand–receptor pairs in biology is usually described by a dissociation constant

(1) $${\text{K}}_{\text{d}}={\text{k}}_{\text{off}}/{\text{k}}_{\text{on}}$$

where k_on_ is the kinetic on rate and k_off_ the kinetic off rate. The more the chemical equilibrium is shifted toward association, *i.e.*, the smaller the value, the stronger the interaction and the more stable the ligand–receptor complex is to stay associated. The k_off_ values for homophilic HFBI hydrophobin interaction are shown in Table 1. The k_off_ values are in the same range despite the interaction environment, *i.e.*, pH or ionic strength. The dissociation rate constant for dimer or multimer formation in solution has not been determined for HFBI, but k_off_ values for HFBII molecules have been reported to be approximately 0.79 s^−1^ in water by Grüner *et al.* (20) by using stopped-flow fluorescence (calculated from the half-life). This value is clearly smaller than for HFBI, but the difference can also originate from the different experimental setup.

**Table 1:** Results of the SMFS experiments performed under different buffer conditions

Buffer	x_β_ [Å]	k_off_ [s^−1^]	n (FDCs)	n (datasets)
PBS	2.85 ± 0.043	3.61 ± 0.015	13,106	7
pH 5 (NaAc)	2.42 ± 0.043	4.44 ± 0.020	13,967	9
H_2_O	2.96 ± 0.039	3.43 ± 0.014	21,096	8

Width of the energy barrier (xβ) and kinetic off-rate (k_off_) were calculated from the FDCs according to Evans theory using a most likelihood approach.

At all the studied conditions the force *versus* logarithm of the loading rate dependencies showed only one linear region (Fig. 3*F*), *i.e.*, only a single energy barrier is present in the energy landscape in HFBI hydrophobin interacting with another HFBI molecule. Also, the x_β_ values for HFBI–HFBI interaction were in the same range despite the environment (Table 1). All these similar results indicate lack of electrostatic participation within the dimerization process between HFBI molecules. Li *et al.* (44) reported the k_off_ value of 0.59 ± 0.16 s^−1^ between HFBI and a hydrophobic surface, and x_β_ of 3.5 ± 0.2 Å. Also, these results support the previously drawn conclusion that the interaction between HFBI and a hydrophobic surface is stronger than between hydrophobin molecules. Moreover, it seems that the hydrophobic patches of HFBI hydrophobins are fully functional in different environments despite the changes in the pH or ionic strength. This, again, supports the dominance of hydrophobic interaction.

Do we now understand better the hydrophobic interaction after measuring interaction forces between hydrophobin molecules at a single molecule level? Hydrophobic interaction is known to be crucial, for example, in micelle formation, in holding protein structures in correct conformation in water, and how the structure of biological membranes is assembled. All these indicate the presence of strong interactions. However, hydrophobin multimers are known to exchange molecules and break at interfaces suggesting that the interaction can be disturbed more easily. The interaction area of hydrophobins can be described as a simple hydrophobic region where association does not require great conformational changes or enclosing of the counter molecule and, thus, can more easily interact with further molecules with hydrophobic regions. This may relate to the fact that the true hydrophobic interactions are short-ranged van der Waals interactions showing relatively strong unbinding forces in force spectroscopy measurements for two hydrophobins being in close contact, whereas in dynamic systems longer-range interactions are able to form more stable complexes. Possibly molecular dynamic simulations of hydrophobin–hydrophobin interaction together with more thorough and systematic force spectroscopy experiments in different pH and ionic strength could reveal more details about the hydrophobic interaction. Maybe they could also help to understand more about the morphology of the hydrophobic patch of hydrophobins and its effect on surrounding water molecules or about the role of hydrophilic residues near the hydrophobic patch. Interaction measurements and simulations on mutated hydrophobin molecules could provide more detailed information about the role of different amino acids in the hydrophobic patch and in the vicinity of the hydrophobic patch. Perhaps all these could reveal the molecular feature that makes the interaction between hydrophobic patches specific but only moderate in strength.

As a summary of this work, the interaction forces between single hydrophobin molecules were determined for the first time. The results confirm that the hydrophobic interaction dominates the interaction and that the hydrophobic interaction is short-ranged. Owing to the short range, the strength of interaction appears moderate in spite of the strong unbinding forces measured and, thus, allows monomer exchange of hydrophobins in solution multimer assemblies. The results also show that hydrophobins are fully functional in different natural environments despite changes in pH or ionic strength. Comparison with recent hydrophobin studies showed that the interaction between a single hydrophobin molecule and a hydrophobic surface is stronger than between two hydrophobin molecules. It is yet unclear what structural features determine this difference; however, it is clear that this behavior has great biological importance for filamentous fungi and the role of hydrophobins in fungal growth and development. The strong surface activity of hydrophobins would not be possible if their assembly to dimers or multimers were stronger than their tendency to attach to surfaces. It is very fascinating, how Nature has evolved something as unique as hydrophobins. Possibly molecular dynamic simulations would reveal more detailed explanation for their behavior.

## Experimental procedures

### Proteins and chemicals

Class II hydrophobin HFBI from *T. reesei* (wildtype, 7.5 kDa) was produced and purified as described (47). Wildtype HFBI was stored freeze-dried. NCys-HFBI (8.7 kDa) is an engineered HFBI variant, which has a cysteine residue in the end of the 11-amino-acid-long linker at the N terminus of the protein (Fig. 1). Production and purification of NCys-HFBI have been described by Szilvay *et al.* (2006) (19). After purification, NCys-HFBI was in the dimeric form, where the introduced sulfhydryl groups form covalent bonds. For surface and tip functionalization NCys-HFBI was selectively reduced into monomers with 50 mM DTT (Sigma-Aldrich) and purified as described (19). The internal disulfides stay intact in these relatively mild reducing conditions (48). After purification, NCys-HFBI stock solution of 1.8 mg/ml was in 0.1% TFA in MilliQ water (pH 1.5) and was stored in 20 μl batches in a freezer (−20 °C). All aliquots were used immediately after thawing.

All chemicals used in experiments were of analytical grade at highest available purity.

### Tip functionalization

Functionalization of commercially available AFM cantilevers (MSCT, nominal spring constants ranging 10–30 pN/nm, Bruker) with NCys-HFBI molecules required three major steps (Fig. 2*A*, upper part). First, after extensive cleaning of the tips, they were aminofunctionalized as described previously, using the gas phase protocol for coupling of APTES (Sigma-Aldrich) (49). For this, two trays, one filled with 60 μl freshly distilled APTES and the other one filled with 20 μl triethylamine (TEA, Sigma-Aldrich) as catalyst, were placed in a 5-l argon-flooded desiccator containing the tips for coating and allowed to react for 120 min. Afterward, APTES and TEA were removed, the desiccator was rinsed extensively with argon gas, and the tips stayed there for 2 days for curing. Second, the generated amino residues were reacted with the heterobifunctional maleimide-PEG_27_-NHS cross-linker (Polypure) in 500 μl chloroform for 120 min (2 mg/ml) using 30 μl TEA (1% v/v) as catalyst yielding in covalently pegylated tips *via* amide bond formation. After washing (3 × 5 min in chloroform) and drying in a gentle stream of nitrogen gas the tips were immediately used for protein coupling. Third the tips were incubated in PBS (150 mM NaCl, 15 nM NaH_2_PO_4_, pH adjusted to 7.4 using NaOH) containing 0.2 mg/ml NCys-HFBI molecules for 60 min resulting in a stable covalent coupling of the hydrophobins *via* their cysteine to the maleimide end of the tip tethered PEG cross-linker (25). To avoid hydrophobin dimers on the tip, the functionalized tips were washed with 70% ethanol before use.

### Functionalization of gold surfaces

A cleaned gold-coated quartz crystal microbalance crystal (Q-Sense) was incubated with 0.2 mg/ml NCys-HFBI molecules for 60 min to allow the formation of a SAM-like layer to gold. For force spectroscopy experiments the samples were rinsed extensively with 70% ethanol in water to remove a possibly formed second hydrophobin layer. All functionalized gold surfaces were used immediately after functionalization.

### Single molecule force spectroscopy

All measurements were performed on a PicoPlus 5500 AFM (Agilent). Hydrophobin-functionalized tips and supports were used as described before. The pulling velocity was varied from 50, 100, 200, 400, 600, 800, 1200, and 2000 nm/s for all different measurement conditions (pure water, 50 mM Na-acetate at pH 5, and PBS buffer pH 7.4). For each pulling velocity and for each of the different conditions 1000 to 3000 FDCs were recorded, except for the very slow pulling speed (50 nm/s: 500–1000 FDCs). The kinetic off-rate constant k_off_ and the distance of the energy barrier from the free equilibrium position x_β_ was determined using a maximum likelihood approach as described (50, 51) and fitting the data according to the single-barrier model (28). In this model the rupture force F∗ is given as a function of the loading rate with

(2) $$\text{F∗}={\text{f}}_{\text{β}}\cdot \ln\left(\text{r}/{\text{k}}_{\text{off}}\cdot {\text{f}}_{\text{β}}\right),$$

where f_β_ is the ratio of the thermal energy k_B_T and x_β_. For comparison of the most probable rupture forces of the different measurement conditions (*i.e.*, pure water, 50 mM Na-acetate at pH 5, and PBS buffer pH 7.4) at the same pulling velocity, PDFs were constructed as described (27). In a PDF, data are weighed by their uncertainty and presented in a continuous curve that allows fitting of a measure of reliability not provided by simple binding. To determine the correct cantilever spring constant the thermal noise method according to Hutter and Bechhoefer (52) was used. The spring constant of every cantilever was measured in triplicates and the mean was used for further data evaluation.

To prove specificity different experimental approaches were used. The “blocking” experiment was performed applying the same experimental setup like in the force spectroscopy experiments but in presence of the native hydrophobin HFBI. For this, wildtype HFBI was added into the liquid cell to yield a final concentration of 0.1 mg/ml. Free HFBI molecules were allowed to adsorb on both the tip and the surface-bound NCys-HFBI for at least 60 min. This was done for PBS, pH 5 and pure water separately. About 1000 to 3000 FDCs at pulling velocities of 500 to 2000 nm/s were performed in each solvent. As a second specificity proof SMFS measurements using the same settings were carried out in 70% ethanol, as according to Szilvay *et al.* (19) higher ethanol concentrations prevent association of hydrophobins.

### AFM imaging

All imaging measurements were performed on a PicoPlus 5500 AFM (Agilent) using cantilever with spring constants of 30 to 100 pN/nm (MSCT, Bruker). Imaging was done in contact mode under near-physiological conditions (PBS, 25 °C). For scratching experiments, the force set point was increased to enhance the indentation force. Scratching was performed four times (2× up and 2× down) on the same area with increased force and speed (16 lines/s), but lowered feedback gains. Both imaging and scratching was done with 512 lines/area. Afterward the force limit was set to a value resulting a low indentation force again, typical for contact mode imaging and the scanning velocity was lowered to one line per second. To image the scratched hole, the scan direction was turned by 90° and the scan range was increased from 500 × 500 nm to 3.6 × 3.6 μm. Quantification of height changes was evaluated by averaging ten cross-sectional profiles using Gwyddion (gwyddion.net).

## Data availability

All data are contained within the article. The raw data will be provided by the corresponding author upon request.

## Conflict of interest

The authors declare that they have no conflicts of interest with the contents of this article.
